# First detection of autochthonous extensively drug-resistant NDM-1 *Pseudomonas aeruginosa* ST235 from a patient with bloodstream infection in Italy, October 2019

**DOI:** 10.1186/s13756-020-00734-5

**Published:** 2020-05-25

**Authors:** Daniela Loconsole, Marisa Accogli, Monica Monaco, Maria Del Grosso, Anna Lisa De Robertis, Anna Morea, Loredana Capozzi, Laura Del Sambro, Annarosa Simone, Vincenzo De Letteriis, Michele Quarto, Antonio Parisi, Maria Chironna

**Affiliations:** 1 Department of Biomedical Sciences and Human Oncology, Hygiene Unit, University of Bari “Aldo Moro”, P.zza G. Cesare 11, 70124 Bari, Italy; 2 Department of Infectious Diseases, Istituto Superiore di Sanità, Roma, Italy; 3 Istituto Zooprofilattico Sperimentale della Puglia e della Basilicata, Foggia, Italy; 4 San Paolo Hospital, ASL, Bari, Italy

**Keywords:** *Pseudomonas aeruginosa*, Extensively-drug resistant, New Delhi metallo beta-, lactamase, Whole-genome sequencing, ST235, sepsis

## Abstract

**Background:**

*Pseudomonas aeruginosa* (PA) is one of the most common and serious causes of healthcare-associated bacteremia. The emergence and dissemination of multidrug-resistant (MDR) and extensively drug-resistant (XDR) PA strains pose a major clinical concern. ST235-PA is a high-risk clone which shows a high capacity to acquire antibiotic resistance. Here we describe the first autochthonous New Delhi metallo-β-lactamase (NDM)-producing *Pseudomonas aeruginosa* ST235 identified in Italy.

**Case presentation:**

In October 2019, a patient residing in an elderly health care and rehabilitation facility, was hospitalized and died from sepsis caused by an XDR-PA. The strain belonged to the high-risk clone sequence type ST235. Whole genome sequencing (WGS) revealed the presence of genes encoding NDM-1 and multiple β-lactamases, many clinically significant multidrug efflux pump complexes and also the virulence gene ExoU, which is associated with a high cytotoxic phenotype.

**Conclusions:**

Few strains of NDM-1-PA have been identified worldwide, all belonging to ST235. The combination of ST235 and ExoU is a predictor of highly unfavorable prognosis. The potential spread of these high-risk clones in healthcare settings is worrisome because treatment options are limited. Early identification of high-risk clones could help in outbreaks investigation and infections control.

## Background

*Pseudomonas aeruginosa* (PA) is an opportunistic human pathogen implicated in various acute and chronic infections, including infections of the respiratory, urinary, and gastrointestinal tracts, as well as bacteremia; it is associated with high mortality rates [1]. Multidrug-resistant *P. aeruginosa* (MDR-PA) and extensively drug-resistant *P. aeruginosa* (XDR-PA) strains are becoming major clinical threats worldwide [2]. Recently, it has been estimated that each year in Italy infections due to antibiotic-resistant bacteria exceed 200,000 cases, causing more than 10,000 deaths. Notably, carbapenem- or colistin-resistant Gram-negative species, including *P. aeruginosa,* contributed considerably to the total burden of antibiotic-resistant infections in health care settings [3]. Data from the European Antimicrobial Resistance Surveillance Network (EARS-Net) showed that in 2018, in Italy, the 15.8% of invasive *P. aeruginosa* isolates were carbapenem-resistant [4]. Moreover, the reported resistance to piperacillin-tazobactam, fluoroquinolones, ceftazidime and aminoglycosides were 23.9%, 22.9%, 19.9% and 12.8%, respectively [4].

Infection with sequence type (ST) 235 has been associated with poor clinical outcomes in patients with *P. aeruginosa* bacteremia [5]. In 2013, a unique New Delhi metallo-β-lactamase-1 (NDM-1)-producing *P. aeruginosa* (NDM-1-PA) ST235 strain was isolated in Italy from a patient with sepsis who had been hospitalized previously in Serbia [6]. In addition, an ST235 NDM-1-PA (HIABP11) was isolated in France in 2012 from the urine culture of a patient hospitalized in Serbia 3 months earlier [7]. The present study describes the first Italian case of an autochthonous extensively drug-resistant *P. aeruginosa* strain producing NDM-1- and β-lactamases isolated from an elderly patient who died from sepsis.

## Case presentation

In August 2019, a 77-year-old woman residing in an elderly health care and rehabilitation facility was hospitalized with chronic respiratory insufficiency caused by chronic obstructive pulmonary disease and pulmonary emphysema in the Internal Medicine ward of San Paolo Hospital in Bari. While hospitalized, she developed a urinary tract infection; *P. aeruginosa* was isolated from a urine sample. No antibiotic was administered. The patient became febrile 3 days later, and *P. aeruginosa* was isolated from blood cultures. She received colistin (a 9 million unit (MU) loading dose, followed by 4.5 MU twice per day) and fosfomycin (6 g every 3 h). Following initial improvement, the patient showed a rapid clinical deterioration and died from sepsis in early November.

The biochemical characteristics and antibiotic susceptibility profile of the *P. aeruginosa* isolate were assessed using the automated VITEK 2 system (bioMérieux, Marcy l’Etoile, France), according to the manufacturer’s instructions. The interpretative breakpoints for minimum inhibitory concentrations (MICs) were based on the criteria of the European Committee on Antimicrobial Susceptibility Testing (EUCAST) (http://www.eucast.org/ast_of_bacteria/). The MIC for colistin was determined using the broth microdilution method, as specified by EUCAST guidelines. The phenotypic resistance patterns of *P. aeruginosa* isolated from urine and blood cultures were identical; except for colistin, all isolates were extensively resistant to all tested antimicrobial agents [8]. Strain serotype, corresponding to O11, was determined using monoclonal antibodies and polyclonal antisera (Bio-Rad Laboratories, Milano, Italy). The main carbapenemase-encoding genes (*bla*_KPC_, *bla*_VIM_, *bla*_NDM_, *bla*_IMP_ and *bla*_OXA-48_) were detected using a commercial multiplex real-time PCR kit (GeneXpert platform, Cepheid, Sunnyvale, CA, USA), which revealed the presence of an NDM gene.

Genomic DNA isolated from a single colony of *P. aeruginosa* was extract using the Qiagen DNeasy Blood and Tissue Kit (Qiagen, Copenaghen, Denmark) and whole-genome sequencing was performed using MiSeq (Illumina San Diego, CA, USA), with a paired-end run of 2 × 250 bp, after Nextera XT paired-end library preparation. De novo genome assembly was performed using SPAdes genome assembler (version 3.12) [9]. The draft genome was approximately 6.941 Mbp in size, with approximately 80× coverage. The multilocus ST was determined by submitting the assembly to the MLST on-line database (https://pubmlst.org/paeruginosa/). The isolate was assigned to ST235 and named UNIBA_ST235PA (GenBank accession number JAABOY000000000).

The resistome and virulome of the genome were analyzed using ABRicate (https://github.com/tseemann/abricate/). With this tool, a BLAST search of genes included in the Resfinder 3.0 database (https://cge.cbs.dtu.dk/services/ResFinder/) and in the Virulence Factors Database (VFDB) (http://www.mgc.ac.cn/VFs/main.htm) was performed on de novo whole-genome assembly.

ResFinder 3.0 analyses showed that the chromosome of the *P. aeruginosa* isolate harbored several antibiotic resistance genes (Table 1). In addition, the isolate encoded many clinically significant multidrug efflux pump complexes belonging to the resistance nodulation cell division family of *P. aeruginosa*.

**Table 1:** Genetic resistance profile of the *P. aeruginosa* NDM-1-producing strain isolated from a patient hospitalized in Bari, Italy, in October 2019

Resistance gene product	Resistance genes
Aminoglycoside modifying enzymes	*aph(3)-IIb, aphA6, aadA6, aac(3)-Ib, aadB, aph(3′)-IIb, aph(3′)-VI, aac(6′)-Ib7, ant(2″)-Ia, aph(3′)-IIb, aph(3′)-VI, aac(6′)-Il, ant(2″)-Ia, aph(3′)-IIb_1, aph(3′)-VI_1, aac(6′)-Il_1, ant(3″)-Ia_1, aadA9*
β-lactamases	*bla*_NDM-1_, *bla*_PAO_, *bla*_OXA-50_, *bla*_OXA-488_, *bla*_PDC-2_, *bla*_PDC-35_
Fluoroquinolone resistance determinant	*crpP*
Macrolide resistance determinant	*ermE*
Bicyclomycin resistance determinant	*bcr-1*
Chloramphenicol resistance determinant	*catB7*
Fosfomycin resistance determinants	*fosA, fosA4*
Triclosan resistance determinants	TriABC-OpmH
Multidrug resistance efflux pumps	MexAB-OprM complex, MexCD-OprJ complex, MexEF-OprN complex, MexGHI-OpmD complex, MexJK complex, MexMN-OprM complex, MexPQ-OpmE complex, MexVW-OprM complex, MexXY-OprM complex, MuxABC-OpmB complex, *armR, arnA, cpxR, pmpM, qacE-H, qacF, soxR*

**Fig. 1:**
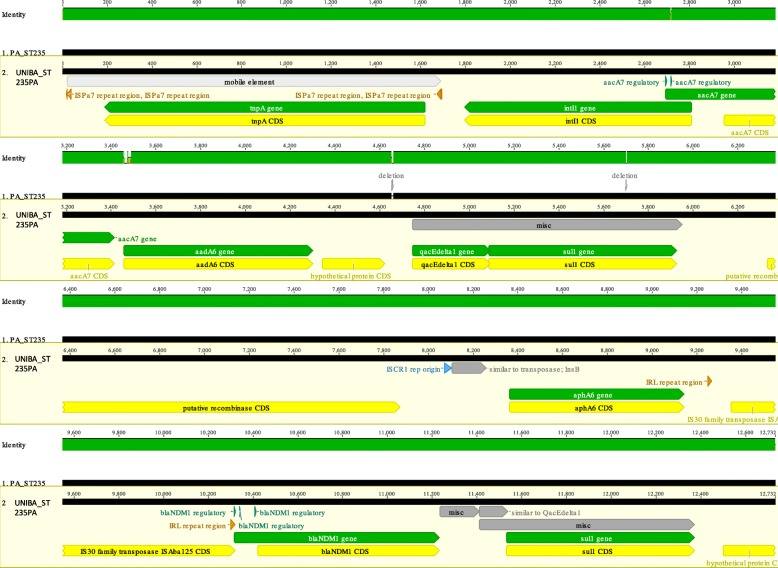
Comparative analysis and genomic representation of the structure of the sequences surrounding the blaNDM-1 gene (12,735 bp) of the *P. aeruginosa* UNIBA_ST235PA strain isolated in this study and of the *P. aeruginosa* HIABPII strain (Acc. no. KC170992)

Comparative genomic analysis of the NDM-1 regions (12,732 bp) of UNIBA_ST235PA and the *P. aeruginosa* HIABP11 strain (Acc. No. KC170992) [7] using Geneious R10.2.6 (Biomatters Ltd., USA) showed that these regions were > 98% identical. The genetic environment surrounding the *bla*_NDM-1_ gene of UNIBA_ST235PA is displayed in the Fig. 1. An IS*CR1* element was located immediately upstream of the chromosomal *bla*_NDM-1_ gene, followed by the aminoglycoside resistance gene *aphA6*; this is identical to that in the HIABP11 strain isolated in France in 2012. In addition, the expression of *bla*_NDM-1_ of UNIBA_ST235PA was under the control of a promoter belonging to the IS*30* family transposase ISA*ba125*. Two short deletions were observed in the mapped sequence of UNIBA_ST235PA: an 8 bp deletion in a non-coding region at position 4564 and a 5 bp deletion at position 5699 in the *sul*1 gene (Fig. 1).

The PlasmidFinder tool (https://cge.cbs.dtu.dk/services/PlasmidFinder/) showed that no plasmids were present in UNIBA_ST235PA.

The virulome of the draft genome identified several virulence determining genes, including the type III secretion system effector ExoU phospholipase, which is associated with a high cytotoxic phenotype, as previously reported [10, 11].

## Discussion and conclusion

Preliminary epidemiologic investigation revealed that the patient had no previous history of travel or hospitalization abroad. Further in-depth epidemiological investigation is ongoing to determine the origin and transmission dynamics of this strain; investigations include the screening of all relatives and personnel involved in the care of this patient. Environmental investigations are also underway. All these activities formed part of public health surveillance. Infection control measures, such as an intensive program of environmental cleaning and disinfection, were implemented to reduce the risk of dissemination of this strain and strict contact isolation precautions were applied. Active surveillance was started to monitor emergence of this pathogen also in patients residing in the healthcare and rehabilitation facility.

To the best of our knowledge, this is the first report describing isolation of an autochthonous, extensively drug-resistant NDM-1 *P. aeruginosa* ST235 strain in Italy. In Italy, most carbapenem-resistant *P. aeruginosa* strains described to date carry the VIM, IMP, and FIM genes [12]. The only NDM-1-carrying PA was isolated from a stem cell transplant recipient previously hospitalized in Belgrade, Serbia [6]. Of note, the genetic environment surrounding the *bla*_NDM-1_ gene of the present isolate was identical to that of the NDM-1-PAs isolated in France in 2012 and in Italy in 2013 [6, 7].

To date, few strains of NDM-1-PA have been identified worldwide; all that have been identified belong to ST235 [12]. ST235 is a high-risk type associated with MDR/XDR profiles of *P. aeruginosa* strains [2]. The XDR phenotype reduces treatment options significantly, potentially deciding the outcome of *P. aeruginosa* infections [5]. Treatment options are limited for patients infected with XDR strains, which impacts the severity and mortality of these infections [5].

Combination chemotherapy, such as ceftazidime-avibactam and ceftazidime-avibactam-fosfomycin, has shown promise for the treatment of MDR/XDR-PA infections [13, 14] but are inactive against metallo-β-lactamases and some OXA-carbapenemases [15].

Unfortunately, the strain described in the present study shows an XDR pattern, which makes therapeutic options even more limited. Moreover, this strain harbored many virulence genes comprising the ExoU, which is associated with clones of serotype O11-ST235 [16]. The combination of ST235 and ExoU is a predictor of highly unfavorable prognosis [5].

In conclusion, the isolation of this autochthonous *P. aeruginosa* ST235 strain encoding genes producing NDM-1- and β-lactamases is worrisome. Early identification of high-risk clones could help in investigating outbreaks and controlling infections. WGS of clinical isolates in particular could allow for a better understanding of the spread of resistance markers, and of possible inter-hospital dissemination of specific clones [2]. Continuous monitoring of the development of antibiotic resistance is crucial if we are to better understand local epidemiology and the potential spread of antimicrobial resistance.

## Data Availability

The nucleotide sequence of UNIBA_ST235PA has been deposited at GenBank under the accession number JAABOY000000000, BioSample SAMN13894995.

## Abbreviations

EUCAST: European Committee on Antimicrobial Susceptibility Testing
MDR: Multidrug-resistant
MICs: Minimum inhibitory concentrations
MU: Million unit
NDM: New Delhi metallo-ß-lactamase
PA: *Pseudomonas aeruginosa*
ST: Sequence type
VFDB: Virulence Factors Database
XDR: Extensively drug-resistant
